# Welcome to a decade of action that can make a change!

**DOI:** 10.1186/1752-2897-7-1

**Published:** 2013-01-28

**Authors:** Uli Schmucker, Axel Ekkernkamp, Dirk Stengel

**Affiliations:** 1 Department Trauma Surgery, University Hospital Greifswald, Road Traffic Crash Research Unit, Sauerbruchstrasse, Germany; 2 Unfallkrankenhaus Berlin, Warener Straße 7, Berlin, 12683, Germany; 3 Department of Trauma and Orthopedic Surgery, Center for Clinical Research, Unfallkrankenhaus Berlin, Warener Str. 7, Berlin, 12683, Germany

## Abstract

The *Journal of Trauma Management and Outcomes* welcomes the launch of the UN Decade of Action for Road Safety 2011–2020. More than 100 countries around the world will kick off the first global Decade of Action for Road Safety 2011–2020, a decade that we believe can make a change!

## 

More than 3,500 people die from road traffic injuries each day [1]. Road traffic injuries are projected to rise from the ninth leading cause of death in 2004 to the fifth in 2030 around the world. Globally, road traffic crashes are the leading cause of death in people aged 15–44 years. Throughout the last decade, most of the progress in road safety has been made in protecting people in cars and people in developed countries. Currently, vulnerable road users account for almost half of the people who are killed in road traffic.

“Today countries and communities are taking action vital to saving lives on our streets and highways” said WHO Director-General Dr. Margaret Chan. “Road traffic crashes are a growing health and development concern affecting all nations, and the Decade offers a framework for an intensified response”. While initiated by the United Nations, the WHO’s task is to coordinate national and international efforts over the Decade and to monitor progress towards achieving the objectives of the Decade [2]. It was estimated, that successful implementation of the proposed global activities could save up to 5 million lives and prevent 50 million serious injuries over the course of the Decade. In this context it was reported that every $1 spent for road safety interventions can save as much as $20 in lost earnings, reduced productivity, and health costs.

Around 90% of the world's road fatalities occur in low-income and middle-income countries, despite the fact that they use only 50% of the world's registered vehicles. The fact that 20 to 30% of the world’s annual fatalities occur in either China or India is striking - though explainable by the rapid motorization and immature trauma systems in these countries.

## Find out if you qualify for our sponsoring program!

In considering road traffic injury a priority global health issue, the *Journal of Trauma Management & Outcomes* declares discretionary support to the objectives of the Decade. In doing so, we offer an unique open-access publication platform to the trauma care and road safety community. Please note that authors from low-income and middle-income countries may qualify for a reduction of the publication fee [3].

We hereby kindly invite you as authors from the trauma care, injury prevention and road safety community to use our online and open-access publication platform. Accepted manuscripts will be published in the new “Decade of Action for Road Safety article series” [4]. The Editorial team welcome any enquiries via jtmo@ukb.de, and we are looking forward to your input.
